# Stochastic SIS Modelling: Coinfection of Two Pathogens in Two-Host Communities

**DOI:** 10.3390/e22010054

**Published:** 2019-12-31

**Authors:** Auwal Abdullahi, Shamarina Shohaimi, Adem Kilicman, Mohd Hafiz Ibrahim, Nader Salari

**Affiliations:** 1 Institute for Mathematical Research, Universiti Putra Malaysia, 43400 Serdang, Selangor, Malaysia; auwal.abdullahi247@gmail.com (A.A.); akilic@upm.edu.my (A.K.); 2 Department of Mathematics and Computer Science, Federal University Kashere, Kashere 771103, Nigeria; 3 Department of Biology, Universiti Putra Malaysia, 43400 Serdang, Selangor, Malaysia; mhafiz_ibrahim@upm.edu.my; 4 Department of Mathematics, Universiti Putra Malaysia, 43400 Serdang, Selangor, Malaysia; 5 Department of Biostatistics, School of Public Health, Kermanshah University of Medical Sciences, 6715847141 Kermanshah, Iran; n.salari@kums.ac.ir

**Keywords:** branching process, continuous time Markov chain, epidemic extinction, Gillespie algorithm, basic reproduction number, stochastic differential equation

## Abstract

A pathogen can infect multiple hosts. For example, zoonotic diseases like rabies often colonize both humans and animals. Meanwhile, a single host can sometimes be infected with many pathogens, such as malaria and meningitis. Therefore, we studied two susceptible classes $S_{1}\left(t\right)$ and $S_{2}\left(t\right)$, each of which can be infected when interacting with two different infectious groups $I_{1}\left(t\right)$ and $I_{2}\left(t\right)$. The stochastic models were formulated through the continuous time Markov chain (CTMC) along with their deterministic analogues. The statistics for the developed model were studied using the multi-type branching process. Since each epidemic class was assumed to transmit only its own type of pathogen, two reproduction numbers were obtained, in addition to the probability-generating functions of offspring. Thus, these, together with the mean number of infections, were used to estimate the probability of extinction. The initial population of infectious classes can influence their probability of extinction. Understanding the disease extinctions and outbreaks could result in rapid intervention by the management for effective control measures.

## 1. Introduction

Animal diseases such as rabies and hantavirus can be transmitted to humans. The pathogens involved therein cause zoonotic epidemics [1]. Since two or more hosts are included in the process, the spread of diseases is sometimes called a multi-host epidemic [2]. For example, rabies is commonly transmitted by domestic dogs [3], and wild rodents play a key role in transmitting hantavirus to humans. Meanwhile, the co-infection of two different pathogens in a single host is common [4]. This can be seen when a host is simultaneously infected by HIV and malaria [5] or by cholera and typhoid [6], for example. Even though many mathematical models describing the dynamics of this process were previously studied [7,8,9], models involving co-transmission and co-infection in multiple hosts are lacking. The probabilities of disease extinction associated with such models are not well-studied. Therefore, this study is concerned with extending the idea of the co-infection of two pathogens to two-host communities such as the zoonotic diseases described above.

In epidemiological studies, in order to capture the infection dynamics of a pathogen spreading into a given host community, two approaches—stochastic and deterministic techniques—are mostly used. As reported in [10,11,12], both of these have their advantages and disadvantages. Though in most scenarios the two techniques are used to analyze a similar problem, their formulations differ. The stochastic models are often derived through continuous time Markov chains (CTMCs); for example, some well-known results obtained by this technique have been used to investigate a variety of problems, such as host–vector malaria in susceptible–infective–susceptible (SIS) and susceptible–infective–recovered (SIR) epidemic models [13]. Meanwhile, the deterministic models formulated through ordinary differential equations (ODEs) can be good at approximating the overall epidemic dynamics when the population size is large [14]; however, when the population is small, stochastic models can be more appropriate to capture the randomness associated with the system [15]. The epidemic extinction can be estimated even if the population is growing exponentially [16]. The differences between stochastic and deterministic models are sometimes determined numerically [17]. When these two models were compared, the stochastic model was able to show that the time until epidemic extinction was longer [18]. Stochastic models have been reported to address some important epidemic questions through parameter estimation on the available data [19].

Another important class of stochastic model is the branching process. For example, a multi-type branching process can be applied to a wide range of epidemic models since it can approximate the CTMC models when answering epidemic questions. Exact solutions to problems of interest are not always available; therefore, asymptotic results derived through the branching process can serve as an alternative means [20]. This can be seen when the asymptotic formulae for the epidemic extinction [21,22], duration of epidemic outbreak [23], and the mean number of infectious individuals [24] were obtained by approximating the CTMC with the multi-type branching process.

A result, the epidemic outbreak is linked with the basic reproduction number [13]. This is an important ratio in epidemiological studies. The management’s intervention, as well as control measures, are concerned with how the epidemic can be minimized within the shortest possible time from the outbreak. This can be achieved when the basic reproduction number is successfully reduced to a threshold value less than one [12]. An epidemic outbreak model incorporating this ratio was used to investigate the stochastic patches, through which the movement of infectious individuals from higher-risk areas to those with lower-risk can serve as another effective control strategy [17]. Similarly, using the basic reproduction number, a stochastic model of two spreading pathogens was studied, through which the effects of key parameters responsible for spreading resistant bacteria in hospitals were determined [25]. The threshold for predicting epidemic extinctions in a single infectious class is ${\left(\frac{1}{R_{0}}\right)}^{x_{0}}$, where $R_{0}$ is the basic reproduction number, and $x_{0}$ is the initial population count of the class [23]. However, this asymptotic result cannot hold for multiple infectious classes [12].

Furthermore, discrete models such as difference equations, which are often used in modeling species with non-overlapping generations (e.g., insects) [26], are not used in this work since the pathogens herein are assumed to be infecting both humans and animals. Therefore, the stochastic models are used to investigate the co-infection of two infectious classes due to their robustness in computing disease extinction. This can be achieved by approximating the multi-type branching process through the transition probabilities. The stochastic model used in this study is of SIS type. The environmental fluctuations of this model were reported to suppress the disease outbreak [27,28,29], determine the coexistence of two infectious diseases [30], and estimate the length of the epidemic disease outbreak [31]. In an attempt to estimate the probability of extinction of two infectious classes co-infecting two-host communities, we (1) derived the deterministic system of ODEs using the transition probabilities of the CTMC models, (2) determined the epidemic extinction and the mean number of infections using asymptotic formulae derived through the multi-type branching process technique, and (3) compared the stochastic models and their deterministic counterparts through numerical simulations.

## 2. Materials and Methods

### 2.1. Continuous Time Markov Chain Model

Suppose that $I_{1}\left(t\right)$ and $I_{2}\left(t\right)$ are population sizes of two infectious classes infecting two susceptible host communities $S_{1}\left(t\right)$ and $S_{2}\left(t\right)$. This can be written as the continuous time Markov chain [32], {$S_{1}\left(t\right),I_{1}\left(t\right),S_{2}\left(t\right),I_{2}\left(t\right);t\geq 0\}$, where *t* is the continuous parameter of the process. Assuming the probability of the population count of the four classes, taking $n_{1},n_{2},n_{3},n_{4}$ is

(1) $$p\left(n_{1},n_{2},n_{3},n_{4};t\right)=p\left(S_{1}\left(t\right)=n_{1},I_{1}\left(t\right)=n_{2},S_{2}\left(t\right)=n_{3},I_{2}\left(t\right)=n4;t\right),$$

whereby $n_{1}=n_{2}=n_{3}=n_{4}=0,1,2,3,....$ Writing the schematic reactions of the given system allows us to derive the deterministic models through the transition probabilities of the CTMC.

1. The rate at which the two infectious classes, $I_{1}\left(t\right)$ and $I_{2}\left(t\right)$, can be recovered are $\Phi_{1}$ and $\Phi_{2}$ respectively,
  $I_{1}\left(t\right)\overset{\Phi_{1}}{\to }\emptyset$ and $I_{2}\left(t\right)\overset{\Phi_{2}}{\to }\emptyset$.
2. The two susceptible classes, $S_{1}\left(t\right)$ and $S_{2}\left(t\right)$, can move to infectious classes, $I_{1}\left(t\right)$ and $I_{2}\left(t\right)$, at the disease transmission rates $\beta_{11},\beta_{12},\beta_{21}$, and $\beta_{22}$, such that
  $S_{1}\left(t\right)+I_{1}\left(t\right)\overset{\beta_{11}}{\to }2I_{1}\left(t\right)$,
  $S_{1}\left(t\right)+I_{2}\left(t\right)\overset{\beta_{12}}{\to }2I_{2}\left(t\right)$,
  $S_{2}\left(t\right)+I_{1}\left(t\right)\overset{\beta_{21}}{\to }2I_{1}\left(t\right)$,
  $S_{2}\left(t\right)+I_{2}\left(t\right)\overset{\beta_{11}}{\to }2I_{2}\left(t\right)$.

Taking the infinitesimal time δt, we list the events happening in the interval (t,t+δt), together with their corresponding transition rates [33] (see Table 1).

Substituting the probabilities given in Table 1, we get the following master equation known as the Kolmogorov forward differential equation:

(2) $$\begin{array}{cc} \frac{dp(n_{1},n_{2},n_{3},n_{4};t)}{dt} & =-p\left(n_{1},n_{2},n_{3},n_{4};t\right)\left(\Phi_{1}n_{2}+\Phi_{2}n_{4}+\beta_{11}n_{1}n_{2}+\beta_{12}n_{1}n_{4}+\beta_{21}n_{2}n_{3}+\beta_{22}n_{3}n_{4}\right)\\ \; & +p\left(n_{1},n_{2}+1,n_{3},n_{4};t\right)\Phi_{1}\left(n_{2}+1\right)\\ \; & +p\left(n_{1},n_{2},n_{3},n_{4}+1;t\right)\Phi_{2}\left(n_{4}+1\right)\\ \; & +p\left(n_{1}+1,n_{2}-1,n_{3},n_{4};t\right)\beta_{11}\left(n_{1}+1\right)\left(n_{2}-1\right)\\ \; & +p\left(n_{1}+1,n_{2},n_{3},n_{4}-1;t\right)\beta_{12}\left(n_{1}+1\right)\left(n_{4}-1\right)\\ \; & +p\left(n_{1},n_{2}-1,n_{3}+1,n_{4};t\right)\beta_{21}\left(n_{2}-1\right)\left(n_{3}+1\right)\\ \; & +p\left(n_{1},n_{2},n_{3}+1,n_{4}-1;t\right)\beta_{22}\left(n_{3}+1\right)\left(n_{4}-1\right). \end{array}$$

The master Equation (2) can be simplified with the aid of the following probability-generating function:

(3) $$F\left(x_{1},x_{2},x_{3},x_{4};t\right)=\sum_{n_{1}}^{\infty }\sum_{n_{2}}^{\infty }\sum_{n_{3}}^{\infty }\sum_{n_{4}}^{\infty }p\left(n_{1},n_{2},n_{3},n_{4};t\right)x_{1}^{n_{1}}x_{1}^{n_{2}}x_{3}^{n_{3}}x_{4}^{n_{4}}.$$

Taking the derivative of Equation (3) with respect to *t* and substituting Equation (2) into the result obtained, we get the following partial differential equation,

(4) $$\begin{array}{cc} \frac{\partial F(x_{1},x_{2},x_{3},x_{4};t)}{\partial t} & =\Phi_{1}\left(1-x_{2}\right)\frac{\partial F(x_{1},x_{2},x_{3},x_{4};t)}{\partial x_{2}}\\ \; & +\Phi_{2}\left(1-x_{4}\right)\frac{\partial F(x_{1},x_{2},x_{3},x_{4};t)}{\partial x_{4}}\\ \; & +\left(\beta_{11}x_{2}\left(x_{2}-x_{1}\right)\right)\frac{\partial^{2}F\left(x_{1},x_{2},x_{3},x_{4};t\right)}{\partial x_{1}\partial x_{2}}\\ \; & +\left(\beta_{12}x_{4}\left(x_{4}-x_{1}\right)\right)\frac{\partial^{2}F\left(x_{1},x_{2},x_{3},x_{4};t\right)}{\partial x_{1}\partial x_{4}}\\ \; & +\left(\beta_{21}x_{2}\left(x_{2}-x_{3}\right)\right)\frac{\partial^{2}F\left(x_{1},x_{2},x_{3},x_{4};t\right)}{\partial x_{2}\partial x_{3}}\\ \; & +\left(\beta_{22}x_{4}\left(x_{4}-x_{3}\right)\right)\frac{\partial^{2}F\left(x_{1},x_{2},x_{3},x_{4};t\right)}{\partial x_{3}\partial x_{4}}. \end{array}$$

Using the moment-generating function $M\left(\Theta_{1},\Theta_{2},\Theta_{3},\Theta_{4};t\right)=F\left(e^{\Theta_{1}},e^{\Theta_{2}},e^{\Theta_{3}},e^{\Theta_{4}}\right)$ [34], Equation (4) can be written as follows:

(5) $$\begin{array}{cc} \frac{\partial M(\Theta_{1},\Theta_{2},\Theta_{3},\Theta_{4};t)}{\partial t} & =\left(\Phi_{1}e^{-\Theta_{2}}-\Phi_{1}\right)\frac{\partial M(\theta_{1},\Theta_{2},\Theta_{3},\Theta_{4};t)}{\partial \Theta_{2}}\\ \; & +\left(\Phi_{2}e^{-\Theta_{4}}-\Phi_{2}\right)\frac{\partial M(\theta_{1},\Theta_{2},\Theta_{3},\Theta_{4};t)}{\partial \Theta_{4}}\\ \; & +\left(\beta_{11}e^{-\Theta_{1}}e^{\Theta_{2}}-\beta_{11}\right)\frac{\partial^{2}M\left(\Theta_{1},\Theta_{2},\Theta_{3},\Theta_{4};t\right)}{\partial \Theta_{1}\partial \Theta_{2}}\\ \; & +\left(\beta_{12}e^{-\Theta_{1}}e^{\Theta_{4}}-\beta_{12}\right)\frac{\partial^{2}M\left(\Theta_{1},\Theta_{2},\Theta_{3},\Theta_{4};t\right)}{\partial \Theta_{1}\partial \Theta_{4}}\\ \; & +\left(\beta_{21}e^{-\Theta_{3}}e^{\Theta_{2}}-\beta_{21}\right)\frac{\partial^{2}M\left(\Theta_{1},\Theta_{2},\Theta_{3},\Theta_{4};t\right)}{\partial \Theta_{2}\partial \Theta_{3}}\\ \; & +\left(\beta_{22}e^{-\Theta_{3}}e^{\Theta_{4}}-\beta_{22}\right)\frac{\partial^{2}M\left(\Theta_{1},\Theta_{2},\Theta_{3},\Theta_{4};t\right)}{\partial \Theta_{3}\partial \Theta_{4}}. \end{array}$$

Given that $\frac{\partial^{r}M\left(0;t\right)}{\partial t}=E\left(X_{i}^{r}\right)$, we obtain $\frac{\partial M(\Theta_{1},\Theta_{2},\Theta_{3},\Theta_{4};t)}{\partial \Theta_{i}}{|}_{\Theta_{i}=0}$ from (5), for i=1,2,3,4, and r=1,2. This gives the following equations:

$$\begin{array}{cc} \frac{dE(X_{1}\left(t\right))}{dt} & =-\beta_{11}E\left(X_{1}\left(t\right)X_{2}\left(t\right)\right)-\beta_{12}E\left(x_{1}\left(t\right)X_{4}\left(t\right)\right)\\ \frac{dE(X_{2}\left(t\right))}{dt} & =-\Phi_{1}E\left(X_{2}\left(t\right)\right)+\beta_{11}E\left(X_{1}\left(t\right)X_{2}\left(t\right)\right)+\beta_{21}E\left(x_{2}\left(t\right)X_{3}\left(t\right)\right)\\ \frac{dE(X_{3}\left(t\right))}{dt} & =-\beta_{21}E\left(X_{2}\left(t\right)X_{3}\left(t\right)\right)-\beta_{22}E\left(X_{3}\left(t\right)X_{4}\left(t\right)\right)\\ \frac{dE(X_{4}\left(t\right))}{dt} & =-\Phi_{2}E\left(X_{4}\left(t\right)\right)+\beta_{12}E\left(X_{2}\left(t\right)X_{4}\left(t\right)\right)+\beta_{22}E\left(X_{3}\left(t\right)X_{4}\left(t\right)\right). \end{array}$$

The heuristic approach allows us to write these equations as follows:

(6) $$\begin{array}{cc} \frac{dS_{1}\left(t\right)}{dt} & =-\left(\beta_{11}I_{1}\left(t\right)+\beta_{12}I_{2}\left(t\right)\right)S_{1}\left(t\right)\\ \frac{dI_{1}\left(t\right)}{dt} & =\left(\beta_{11}S_{1}\left(t\right)+\beta_{21}S_{2}\left(t\right)\right)I_{1}\left(t\right)-\Phi_{1}I_{1}\left(t\right)\\ \frac{dS_{2}\left(t\right)}{dt} & =-\left(\beta_{21}I_{1}\left(t\right)+\beta_{22}I_{2}\left(t\right)\right)S_{2}\left(t\right)\\ \frac{dI_{2}\left(t\right)}{dt} & =\left(\beta_{12}S_{1}\left(t\right)+\beta_{22}S_{2}\left(t\right)\right)I_{2}\left(t\right)-\Phi_{2}I_{2}\left(t\right). \end{array}$$

The compartmental differential Equations (6) describe the dynamics of two infectious classes $I_{1}\left(t\right)$ and $I_{2}\left(t\right)$ mixing with two susceptible groups $S_{1}\left(t\right)$ and $S_{2}\left(t\right)$. The birth and immigration of individuals are not captured by the model.

If the recovery rates, $\Phi_{1}$ and $\Phi_{2}$, are not considered in the process, we obtain the following master equation:

(7) $$\begin{array}{cc} \frac{dp(n_{1},n_{2},n_{3},n_{4};t)}{dt} & =-p\left(n_{1},n_{2},n_{3},n_{4};t\right)\left(\beta_{11}n_{1}n_{2}+\beta_{12}n_{1}n_{4}+\beta_{21}n_{2}n_{3}+\beta_{22}n_{3}n_{4}\right)\\ \; & +p\left(n_{1}+1,n_{2}-1,n_{3},n_{4};t\right)\beta_{11}\left(n_{1}+1\right)\left(n_{2}-1\right)\\ \; & +p\left(n_{1}+1,n_{2},n_{3},n_{4}-1;t\right)\beta_{12}\left(n_{1}+1\right)\left(n_{4}-1\right)\\ \; & +p\left(n_{1},n_{2}-1,n_{3}+1,n_{4};t\right)\beta_{21}\left(n_{2}-1\right)\left(n_{3}+1\right)\\ \; & +p\left(n_{1},n_{2},n_{3}+1,n_{4}-1;t\right)\beta_{22}\left(n_{3}+1\right)\left(n_{4}-1\right). \end{array}$$

Applying similar techniques to Equation (7), we get the following system of ordinary differential equations:

(8) $$\begin{array}{cc} \frac{dS_{1}\left(t\right)}{dt} & =-\left(\beta_{11}I_{1}\left(t\right)+\beta_{12}I_{2}\left(t\right)\right)S_{1}\left(t\right)\\ \frac{dI_{1}\left(t\right)}{dt} & =\left(\beta_{11}S_{1}\left(t\right)+\beta_{21}S_{2}\left(t\right)\right)I_{1}\left(t\right)\\ \frac{dS_{2}\left(t\right)}{dt} & =-\left(\beta_{21}I_{1}\left(t\right)+\beta_{22}I_{2}\left(t\right)\right)S_{2}\left(t\right)\\ \frac{dI_{2}\left(t\right)}{dt} & =\left(\beta_{12}S_{1}\left(t\right)+\beta_{22}S_{2}\left(t\right)\right)I_{2}\left(t\right). \end{array}$$

Thus, Equations (8) describe the dynamics of two infectious classes excluding the recovery terms in the disease compartments.

### 2.2. Basic Reproduction Number

As introduced earlier, the basic reproduction number is an important threshold for measuring how diseases are spreading in communities. When determining the basic reproduction number of the derived model (6), we used the matrix $\rho \left(K\right)=FV^{-1}$, which is referred to as the next-generation matrix. While *F* represents the matrix of infection rates, *V* is that of the transfer rates in the disease compartments. These can be analytically obtained by linearizing the system of ordinary differential equations near the disease free-equilibrium [21,35] as follows:

$$F=\left[\begin{array}{cc} \beta_{11}+\beta_{21} & 0\\ 0 & \beta_{12}+\beta_{22} \end{array}\right]$$

and

$$V=\left[\begin{array}{cc} \Phi_{1} & 0\\ 0 & \Phi_{2} \end{array}\right].$$

The spectral radius of the next-generation matrix ρ(K) gives the following results [4,36]:

(9) $$R_{01}=\frac{\beta_{11}+\beta_{21}}{\Phi_{1}}$$

and

(10) $$R_{01}=\frac{\beta_{12}+\beta_{22}}{\Phi_{2}}.$$

Therefore, the basic reproduction number associated with the system can be written as

(11) $$R_{0}=\max\left\{R_{01},R_{02}\right\}.$$

### 2.3. Multi-Type Branching Process

To examine the probability of epidemic extinction for the system of ODE Equations (6), we employed the multi-type branching process. An important theorem for determining such probabilities along with other statistics is stated as follows.

**Theorem** **1.** *Suppose that the function $G\left(s_{1},s_{2},...,s_{h}\right)=\left(g_{1}\left(s_{1},s_{2},...,s_{h}\right),...,g_{n}\left(s_{1},s_{2},...,s_{h}\right)\right)$, with non-linear variables $s_{1},s_{2},...,s_{n}$, is the probability-generating function of offspring, whose mean number is represented by matrix M. If the dominant eigenvalue of M λ≤1, then*

$$lim_{n\to \infty }Prob\left\{Z\left(n\right)=0|Z\left(0\right)=\delta_{j}\right\}=1,$$

*whereby j=1,2,...,h. A unique vector $w=w_{1},w_{2},...w_{h}$ can exist only if λ>1 for $0<w_{j}<1$, j=1,2,...,h such that*

$$lim_{n\to \infty }Prob\left\{Z\left(n\right)=0|Z\left(0\right)=\delta_{j}\right\}=w_{j},$$

*whereby $w_{j}$ represents the fixed point generated by $g_{h}\left(w_{1},w_{2},...w_{n}\right)$, and $\delta_{j}$ is a Kronecker delta.*

This theorem guarantees that we can determine the fixed points of equations of offspring as well as their mean numbers through the multi-step branching process. This process could be used to approximate our CTMC model. We assumed that $\Omega \left(t\right)=(\Omega_{1}\left(t\right),\Omega_{2}\left(t\right),\Omega_{3}\left(t\right),\Omega_{4}\left(t\right))$ represents the four classes of discrete random variables $\left(S_{1}\left(t\right),I_{1}\left(t\right),S_{2}\left(t\right),I_{2}\left(t\right)\right)$. Two classes of interest, through which the epidemic extinction and outbreak can be estimated, are $\Omega_{2}\left(t\right)$ and $\Omega_{4}\left(t\right)$.

The probability of the epidemic extinction for the two classes $I_{1}\left(t\right)$ and $I_{2}\left(t\right)$ can be computed by taking the probability-generating function of the two random variables $\Omega_{2}\left(t\right)$ and $\Omega_{4}\left(t\right)$,

(12) $$g_{1}\left(s_{1},s_{2}\right)=\frac{\beta_{11}s_{1}^{2}+\beta_{21}s_{1}^{2}+\Phi_{1}}{\beta_{11}+\beta_{21}+\Phi_{1}},$$

(13) $$g_{2}\left(s_{1},s_{2}\right)=\frac{\beta_{12}s_{2}^{2}+\beta_{22}s_{2}^{2}+\Phi_{2}}{\beta_{12}+\beta_{22}+\Phi_{2}}.$$

Considering the transition rates for the CTMC model in Table 1, we notice that the individual of type 1 can only give birth to its own type. This is similar to the individual of type 2.

The mean number of infections can be determined using the formula [24]:

$$M=\frac{\partial g_{1,2}\left(s_{1},s_{2}\right)}{\partial s_{j}}{|}_{s_{1}=1,s_{2}=1,j=1,2}$$

as

$$M=\left[\begin{array}{cc} \frac{2(\beta_{11}+\beta_{21})}{\beta_{11}+\beta_{21}+\Phi_{1}} & 0\\ \frac{2(\beta_{12}+\beta_{22})}{\beta_{12}+\beta_{22}+\Phi_{2}} & 0 \end{array}\right].$$

The probability of epidemic extinction for the system of ODEs (6) can be obtained since matrix *M* is consistent with the basic reproduction number $R_{0}$ (11). For the sub-critical case, the probability can be investigated by applying the jury condition [37], which states that the spectral radius of matrix *M*, ρ(M)<1 if and only if

trace(M)<1+det(M)<2.

It is clear from matrix *M*,

$$\mathrm{trace}\left(M\right)=\frac{2(\beta_{11}+\beta_{21})}{\left(\beta_{11}+\beta_{21}+\Phi_{1}\right)}>0,$$

and det(M)=0<1. Thus, the jury condition holds for matrix *M*, and this also satisfies the first part of Theorem 1.

Since matrix *M* is reducible, the epidemic extinction in the super-critical case can be determined by considering the two basic reproduction numbers $R_{01}$ and $R_{02}$. Solving for $g_{1}\left(s_{1},s_{2}\right)=s_{1}$ and $g_{2}\left(s_{1},s_{2}\right)=s_{2}$ in (12),(13), we get $s_{1}=s_{2}=1$, $s_{1}=\frac{\Phi_{1}}{\beta_{11}+\beta_{21}}$, and $s_{2}=\frac{\Phi_{2}}{\beta_{12}+\beta_{22}}$. While the epidemic transmission and its recovery rates for $I_{1}\left(t\right)$ are $\beta_{11}+\beta_{21}$ and $\Phi_{1}$, respectively, those of $I_{2}\left(t\right)$ can be written as $\beta_{12}+\beta_{22}$ and $\Phi_{2}$, respectively. Thus, the fixed points $\left(w_{1},w_{2}\right)\in {\left(0,1\right)}^{2}$ for the probability-generating functions of the offspring, and the two non-zero entries $M_{1}=\frac{2(\beta_{11}+\beta_{21})}{\beta_{11}+\beta_{21}+\Phi_{1}}$ and $M_{2}=\frac{2(\beta_{12}+\beta_{22})}{\beta_{12}+\beta_{22}+\Phi_{2}}$ of matrix *M* can be used as follows:

The epidemic extinction in the super-critical case can be determined if $R_{01}>1$, $\rho (M_{1})>1$, the fixed point is

(14) $$w_{1}=\left(\frac{1}{R_{01}}\right).$$

Similarly, if $R_{02}>1$, $\rho (M_{2})>1$, we apply the fixed point

(15) $$w_{2}=\left(\frac{1}{R_{02}}\right).$$

These satisfy the second part of Theorem 1. Since each infectious class transmits only its own type of pathogen, the determination of inverses for the two basic reproduction numbers (Equations (14), (15)) allows us to write the probabilities of extinction for the two infectious classes separately. Thus, for $I_{1}\left(0\right)=x_{0}$, we obtained the following [16,23]:

$$P{}_{01}=\left\{\begin{array}{cc} w_{1}^{x_{0}} & R_{01}>1\\ 1 & R_{01}<1. \end{array}\right.$$

Meanwhile, the epidemic extinction of the second class for $I_{2}\left(0\right)=y_{0}$ can be written as

$$P{}_{02}=\left\{\begin{array}{cc} w_{2}^{y_{0}} & R_{02}>1\\ 1 & R_{02}<1. \end{array}\right.$$

A minor outbreak or epidemic extinction occurs if $R_{01}<1$, $R_{02}<1$, whereas a major outbreak can exist only if $R_{01}>1$, $R_{02}>1$ [13].

## 3. Results

### Numerical Examples

Since it can be difficult to analytically solve the transition probabilities from the forward Kolmogorov Equations (2) and (7), the Gillespie realizations conveniently give their numerical approximations. This can be achieved by drawing two pseudo-random numbers between zero and one from the uniform distribution. While the first random number allows jumps to the next states, the second is the inter-event time representing the time elapsing between events in the process [38]. Each stochastic realization is obtained with the successive number of these steps.

**Figure 1:**
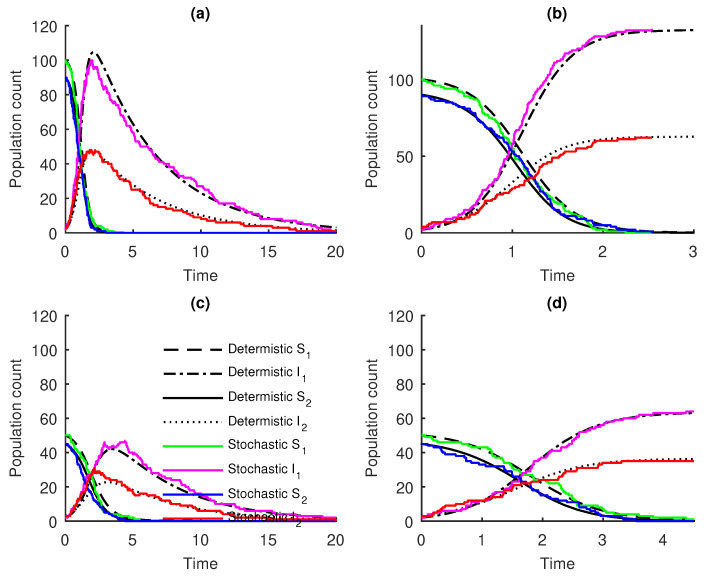
One stochastic realisation approximates the deterministic models for $\beta_{11}=0.02$, $\beta_{12}=0.01$, $\beta_{21}=0.02$, $\beta_{22}=0.02$, given (**a**) the sample paths of Equations (6) with $\Phi_{1}=\Phi_{2}=0.2$, $S_{1}\left(0\right)=100$, $I_{1}\left(0\right)=2$, $S_{2}\left(0\right)=90$, and $I_{2}\left(0\right)=3$; (**b**) the sample paths of Equations (8) with the initial conditions $S_{1}\left(0\right)=100$, $I_{1}\left(0\right)=2$, $S_{2}\left(0\right)=90$, and $I_{2}\left(0\right)=3$; (**c**) the sample paths of Equations (6) for $\Phi_{1}=\Phi_{2}=0.2$, $S_{1}\left(0\right)=50$, $I_{1}\left(0\right)=2$, $S_{2}\left(0\right)=45$, and $I_{2}\left(0\right)=3$; and (**d**) the sample paths of Equations (8) with the initial conditions $S_{1}\left(0\right)=50$, $I_{1}\left(0\right)=2$, $S_{2}\left(0\right)=45$, and $I_{2}\left(0\right)=3$.

The stochastic model was found to agree with its deterministic counterpart. Using arbitrary parameters, the disease transmission rates, $\beta_{11}=\beta_{21}=\beta_{22}=0.02$, $\beta_{12}=0.01$, the infectious recovery rates $\Phi_{1}=\Phi_{2}=0.2$, the initial number of susceptible individuals $S_{1}\left(0\right)=100$, $S_{2}\left(0\right)=90$, and the initial number of infectious classes, $I_{1}\left(0\right)=2$, $I_{2}\left(0\right)=3$, one Gillespie realization from each class approximately converges with their deterministic analogues (see Figure 1a,c). Though any other suitable values can be used to test the dynamics of the two infectious classes, the infection rates $\beta_{11},\beta_{12},\beta_{21},and\beta_{22}$ were held constant to allow us to examine the changes in $I_{1}\left(t\right)$ and $I_{2}\left(t\right)$ when varying the initial conditions of the two host communities. Thus, reducing the population of susceptible individuals affects the number of each infectious class (Figure 1). Removing the recovery terms in disease compartments (Equations (8)), we observed the disease persistence in the system (see Figure 1b,c).

**Figure 2:**
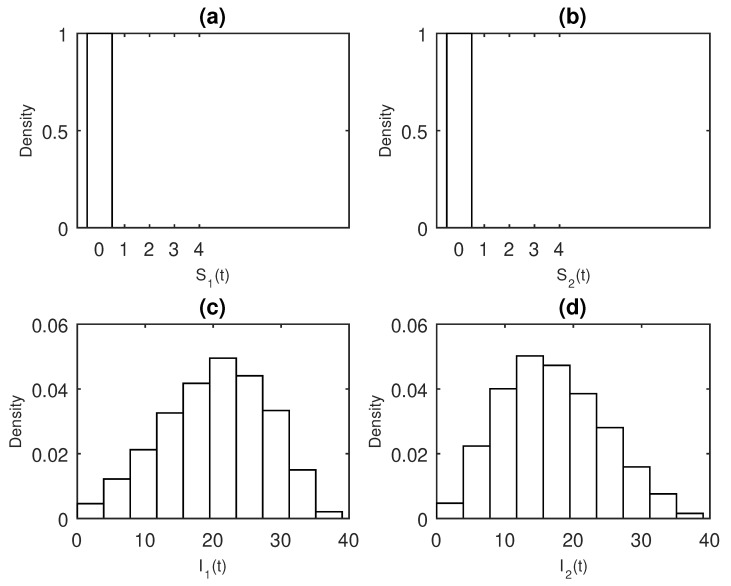
Solutions of the compartments, (**a**) $S_{1}\left(t\right)$, (**b**) $S_{2}\left(t\right)$, (**c**) $I_{1}\left(t\right)$, (**d**) $I_{2}\left(t\right)$, of the epidemic model (6) at t=20 based on 1000 stochastic realisations. The parameters used for determining the distributions are $\beta_{11}=0.02$, $\beta_{12}=0.01$, $\beta_{21}=0.02$, $\beta_{22}=0.02$, $\Phi_{1}=\Phi_{2}=0.2$, $S_{1}\left(0\right)=100$, $I_{1}\left(0\right)=2$, $S_{2}\left(0\right)=90$, and $I_{2}\left(0\right)=3$.

To examine the behavior of the stochastic realizations after a long period of time, the distribution of the process can serve as an alternative solution. Using the parameters described above, $\beta_{11}=\beta_{21}=\beta_{22}=0.02$, $\beta_{12}=0.01$ $\Phi_{1}=\Phi_{2}=0.2$, $S_{1}\left(0\right)=100$, $S_{2}\left(0\right)=90$, $I_{1}\left(0\right)=2$, $I_{2}\left(0\right)=3$, one can see that at t=20, the population of infectious individuals is nearly extinct (Figure 2). This indicates that both Figure 1 and Figure 2 capture the dynamics of the co-infection models, Equations (6) and (8).

To determine the probability of extinction of each infectious class, we employed both the asymptotic formulae P${}_{01}$ and P${}_{02}$, as well as the CTMC simulations. Though not all the entries of Table 2 were the same for both P${}_{01}$, P${}_{02}$ and CTMC simulations (Approx 1 and Approx 2), they reveal that the extinction of each infectious class depended on its initial population size, when their numbers were small [21]. This is because the probability of disease extinction decreases with the increase in the initial population size of each of the two infectious classes (see Table 2).

## 4. Discussion

In this study, the probability of epidemic extinction of two infectious classes co-infecting two-host communities was estimated. The basic reproduction number, a threshold for determining epidemic extinction, can be applied to a single infectious class to study problems related to probabilities of extinctions and outbreaks [23]. However, when many infectious classes are to be studied, their probabilities of extinction can be estimated through the matrix of mean number of infections obtained from the multi-type branching process [16]. When determining this result in this work, we reported a reducible matrix. Therefore, no single explicit formula could be used to estimate the extinctions of the two infectious classes related to our formulation (Equation (6)).

Furthermore, since the transition probabilities for our CTMC model (Table 1) were used when formulating the probability-generating functions of the offspring for the multi-type branching process, our study reported the emergence of two basic reproduction numbers, $R_{01}$ and $R_{02}$, for the two infectious classes $I_{1}\left(t\right)$ and $I_{2}\left(t\right)$, respectively. This is consistent with what has been obtained using the next-generation matrix in this study and others [4,12,36]. Using the two basic reproduction numbers and CTMC simulations, the epidemic extinctions of two infectious classes which infect two-host communities was estimated. Our results show that the CTMC simulations could nearly approximate the asymptotic results involving reproduction numbers when many sample paths were used (i.e., $10^{6}$). This is similar to what was reported in [16]. Our results equally show that the extinction of each infectious class depended on the initial number of individuals therein (Table 2).

The Gillespie realizations were reported to approximate the deterministic model (see Figure 1). This is similar to what has been reported in many epidemiological studies comparing stochastic and deterministic models, such as [11,18]. When comparing the solutions of models (6) and (8), our results showed that the disease persistence could be suppressed by introducing recovery terms in the disease compartments.

## 5. Conclusions

The formulation and analyses of the stochastic models of two infectious classes co-infecting two different susceptible groups can give additional information on how pathogens spread in host communities. Estimation of the disease extinction through the multi-type branching process supplements our results obtained by the deterministic model, such as the basic reproduction number.

Specifically, the stochastic formulation of SIS models provides essential information on the extinctions of two pathogens spreading in two host communities. The transition rates of the CTMC not only produce the deterministic system of ODEs through the master equations, but also approximate those (transition rates) of the multi-type branching process. This provides information about the disease extinction using a small number of initial population sizes of the infectious classes. Both the stochastic SIS simulations and the solutions of ODEs suggest that removing the recovery terms in the disease compartments can lead to the persistence of diseases.

## Figures and Tables

**Table 1:** Assumptions of the $S_{1}I_{1}S_{2}I_{2}$ continuous time Markov chain (CTMC) model.

Event	Transition Between *t* and t+δt	Probability
(i) Mortality of $I_{1}\left(t\right)$	$\left(n_{1},n_{2}+1,n_{3},n_{4}\right)$→$\left(n_{1},n_{2},n_{3},n_{4}\right)$	$\Phi_{1}\left(n_{2}+1\right)\delta t+0{\left(\delta t\right)}^{2}$
(ii) Mortality of $I_{2}\left(t\right)$	$\left(n_{1},n_{2},n_{3},n_{4}+1\right)$→$\left(n_{1},n_{2},n_{3},n_{4}\right)$	$\Phi_{2}\left(n_{4}+1\right)\delta t+0{\left(\delta t\right)}^{2}$
(iii)$I_{1}\left(t\right)$ infects $S_{1}\left(t\right)$	$\left(n_{1}+1,n_{2}-1,n_{3},n_{4}\right)$→$\left(n_{1},n_{2},n_{3},n_{4}\right)$	$\beta_{11}\left(n_{1}+1\right)\left(n_{2}-1\right)\delta t+0{\left(\delta t\right)}^{2}$
(iv)$I_{2}\left(t\right)$ infects $S_{1}\left(t\right)$	$\left(n_{1}+1,n_{2},n_{3},n_{4}-1\right)$→$\left(n_{1},n_{2},n_{3},n_{4}\right)$	$\beta_{12}\left(n_{1}+1\right)\left(n_{4}-1\right)\delta t+0{\left(\delta t\right)}^{2}$
(v)$I_{1}\left(t\right)$ infects $S_{2}\left(t\right)$	$\left(n_{1},n_{2}-1,n_{3}+1,n_{4}\right)$→$\left(n_{1},n_{2},n_{3},n_{4}\right)$	$\beta_{21}\left(n_{2}-1\right)\left(n_{3}+1\right)\delta t+0{\left(\delta t\right)}^{2}$
(vi)$I_{2}\left(t\right)$ infects $S_{2}\left(t\right)$	$\left(n_{1},n_{2},n_{3}+1,n_{4}-1\right)$→$\left(n_{1},n_{2},n_{3},n_{4}\right)$	$\beta_{22}\left(n_{3}+1\right)\left(n_{4}-1\right)\delta t+0{\left(\delta t\right)}^{2}$

**Table 2:** The probabilities of extinction P${}_{01}$ and P${}_{02}$ of two infectious classes $I_{1}\left(t\right)$ and $I_{2}\left(t\right)$, while Approx 1 and 2 are their respective numerical estimations using CTMC for $\beta_{11}=\beta_{12}=\beta_{21}=\beta_{22}=0.2$, $\Phi_{1}$ = $\Phi_{2}$ = 0.3 based on $10^{6}$ sample paths.

$I_{1}\left(0\right)$	$I_{2}\left(0\right)$	P${}_{01}$	Approx 1	P${}_{02}$	Approx 2
1	1	0.7500	0.4997	0.7500	0.5003
2	1	0.5625	0.3333	0.7500	0.6667
1	2	0.7500	0.6664	0.5625	0.3336
2	2	0.5625	0.5001	0.5625	0.4999
3	2	0.4219	0.3995	0.5625	0.6004
2	3	0.5625	0.6000	0.4219	0.4000
3	3	0.5000	0.5000	0.4219	0.4219
